# A Stabilized, Monomeric, Receptor Binding Domain Elicits High-Titer Neutralizing Antibodies Against All SARS-CoV-2 Variants of Concern

**DOI:** 10.3389/fimmu.2021.765211

**Published:** 2021-12-09

**Authors:** Shahbaz Ahmed, Mohammad Suhail Khan, Savitha Gayathri, Randhir Singh, Sahil Kumar, Unnatiben Rajeshbhai Patel, Sameer Kumar Malladi, Raju S. Rajmani, Petrus Jansen van Vuren, Shane Riddell, Sarah Goldie, Nidhi Girish, Poorvi Reddy, Aditya Upadhyaya, Suman Pandey, Samreen Siddiqui, Akansha Tyagi, Sujeet Jha, Rajesh Pandey, Oyahida Khatun, Rohan Narayan, Shashank Tripathi, Alexander J. McAuley, Nagendrakumar Balasubramanian Singanallur, Seshadri S. Vasan, Rajesh P. Ringe, Raghavan Varadarajan

**Affiliations:** ^1^ Molecular Biophysics Unit (MBU), Indian Institute of Science, Bengaluru, India; ^2^ Mynvax Private Limited, ES12, Entrepreneurship Centre, Society for Innovation and Development (SID), Indian Institute of Science, Bengaluru, India; ^3^ Virology Unit, Institute of Microbial Technology, Council of Scientific and Industrial Research (CSIR), Chandigarh, India; ^4^ Australian Centre for Disease Preparedness (ACDP), Commonwealth Scientific and Industrial Research Organisation (CSIRO), Geelong, VIC, Australia; ^5^ Max Super Speciality Hospital (A Unit of Devki Devi Foundation), Max Healthcare, Delhi, India; ^6^ INtegrative GENomics of HOst-PathogEn (INGEN-HOPE) Laboratory, Council of Scientific and Industrial Research (CSIR)-Institute of Genomics and Integrative Biology (CSIR-IGIB), Delhi, India; ^7^ Department of Microbiology & Cell Biology, Indian Institute of Science, Bengaluru, India; ^8^ Centre for Infectious Disease Research, Indian Institute of Science, Bengaluru, India; ^9^ Department of Health Sciences, University of York, York, United Kingdom

**Keywords:** stabilizing mutation, hyperstable mutants, neutralizing antibodies, hamster immunization, vaccination, lyophilization, thermotolerance

## Abstract

Saturation suppressor mutagenesis was used to generate thermostable mutants of the SARS-CoV-2 spike receptor-binding domain (RBD). A triple mutant with an increase in thermal melting temperature of ~7°C with respect to the wild-type B.1 RBD and was expressed in high yield in both mammalian cells and the microbial host, *Pichia pastoris*, was downselected for immunogenicity studies. An additional derivative with three additional mutations from the B.1.351 (beta) isolate was also introduced into this background. Lyophilized proteins were resistant to high-temperature exposure and could be stored for over a month at 37°C. In mice and hamsters, squalene-in-water emulsion (SWE) adjuvanted formulations of the B.1-stabilized RBD were considerably more immunogenic than RBD lacking the stabilizing mutations and elicited antibodies that neutralized all four current variants of concern with similar neutralization titers. However, sera from mice immunized with the stabilized B.1.351 derivative showed significantly decreased neutralization titers exclusively against the B.1.617.2 (delta) VOC. A cocktail comprising stabilized B.1 and B.1.351 RBDs elicited antibodies with qualitatively improved neutralization titers and breadth relative to those immunized solely with either immunogen. Immunized hamsters were protected from high-dose viral challenge. Such vaccine formulations can be rapidly and cheaply produced, lack extraneous tags or additional components, and can be stored at room temperature. They are a useful modality to combat COVID-19, especially in remote and low-resource settings.

## Introduction

SARS-CoV-2 is the etiological agent of the ongoing pandemic which has so far resulted in over four million deaths worldwide (1). The trimeric spike glycoprotein is the major surface protein of SARS-CoV-2 and the principal target of neutralizing antibodies (2). Each protomer consists of two subunits, S1 and S2 (3). The S1 subunit contains the receptor-binding domain (RBD) which interacts with the ACE-2 receptor and mediates viral entry *via* the fusion machinery present in the S2 subunit (4). The RBD is the major target of the neutralizing response (5–7), and serum titers against the RBD correlate well with neutralization titers. There are currently several vaccines in preclinical development and clinical use which include viral vector vaccines (8–14), nucleic acid vaccines (15–17), and inactivated virus vaccines (18, 19). A recombinant subunit vaccine candidate Novavax (NVX-CoV2373) (20) has also shown promising results. Of the vaccines currently in clinical use, the two mRNA vaccines have the highest clinical efficacy but also require ultra-low-temperature storage and are more expensive than the other modalities. The immunogens currently in clinical use or clinical trials are derived from the wild-type (WT) sequence (21) and employ the full-length viral spike as the primary antigen. In recent months, several viral variants of concern (VOC) have emerged. Current VOC are B.1.1.7 (alpha), B.1.351 (beta), P.1 (gamma), and B.1.617.2 (delta). Both B.1.351 and B.1.617.2 show substantially decreased neutralization by many existing monoclonal antibodies and by convalescent as well as vaccine sera (22–24). The B.1.617.2 variant is currently driving a worldwide resurgence in infections (25–27). Thus, there is still a need for inexpensive, rapidly producible, and highly efficacious vaccines against VOC, which preferably do not require low-temperature storage. We recently showed that both monomeric and intermolecular disulfide-linked, trimeric RBD derivatives were highly thermotolerant with the latter showing improved immunogenicity (28), albeit with significant antibody titers against the trimerization domain.

**Figure 1:**
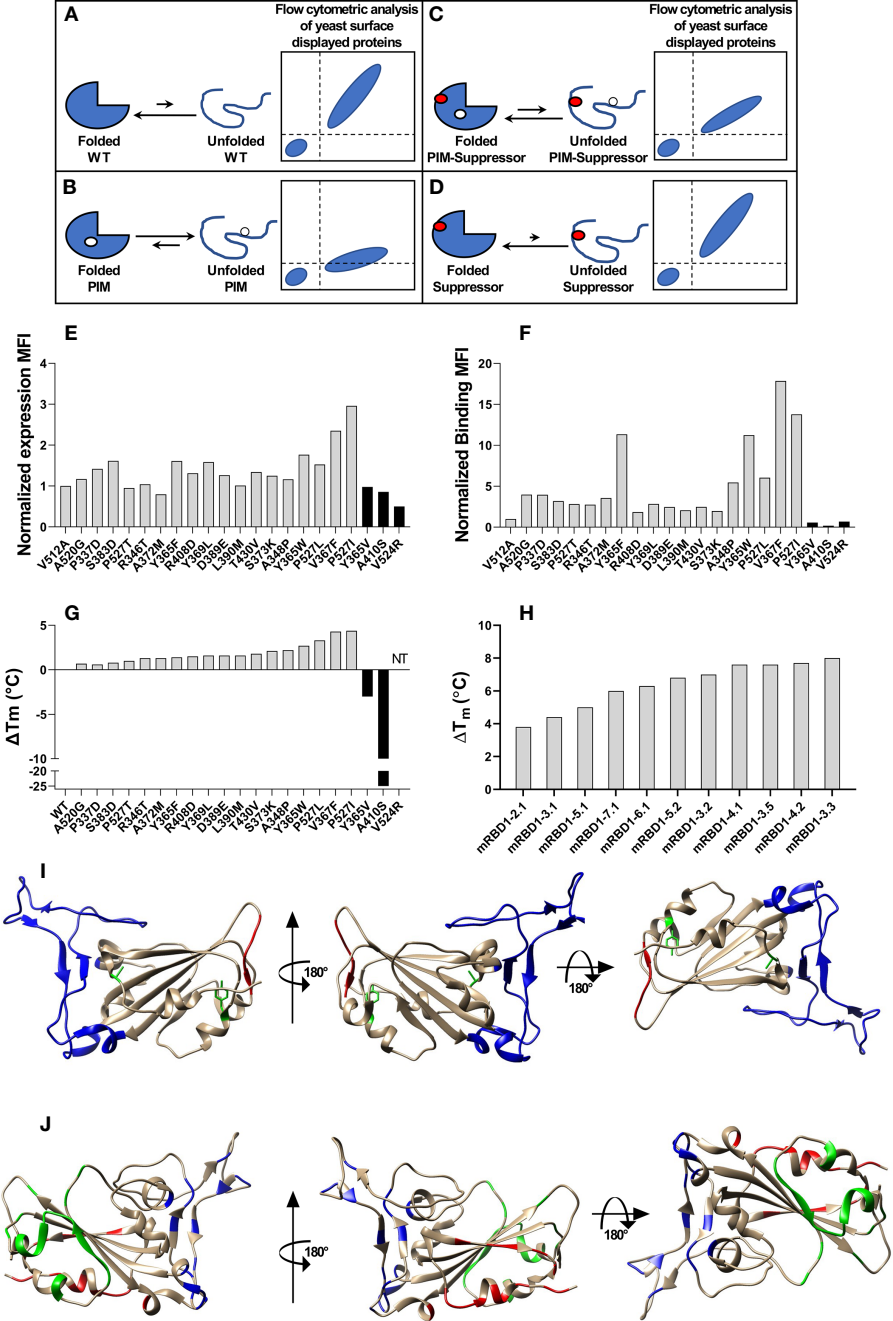
Stabilized mutant identification using second-site saturation suppressor mutagenesis (SSSM). Schematic representation of second-site saturation suppressor mutagenesis. Proteins exist in an equilibrium between folded and unfolded states. **(A)** Generally, in the case of WT proteins, equilibrium is shifted toward the folded conformation. Such proteins show high levels of folded expressed protein when expressed on the yeast cell surface and bound to their cognate partner. However, upon introduction of a **(B)** parent inactive mutation (PIM), the equilibrium is shifted toward the unfolded state and the extent of equilibrium shift will be determined by the destabilizing effect of the PIM. Such PIMs show a lower level of expressed as well as folded proteins on the yeast cell surface. **(C)** The equilibrium between folded and unfolded states of the PIM can be shifted toward the folded state if a suppressor mutation is introduced. Such double mutants show higher levels of folded expressed proteins on yeast cell surface compared to the PIM alone. **(D)** Such suppressor mutants generally have a higher amount of folded fraction at the equilibrium than the WT protein. However, the amount of expressed protein on the yeast cell surface will be similar to WT. Normalized MFI of suppressor mutant **(E)** expression and **(F)** binding of individually analyzed putative suppressors in the background of the PIM. The MFI of double mutants was normalized with the MFI of PIM, and a twofold cutoff was used to differentiate between putative suppressors and non-suppressor mutations. **(G)** ΔT_m_ of purified single mutants identified from suppressor analysis, measured by nano-DSF. The mutants were categorized into stabilized, WT-like, and destabilized mutants indicated by light gray, dark gray, and back bars, respectively. Binding MFI of double mutants relative to PIM **(E)** robustly identifies stabilizing suppressors. **(H)** ΔT_m_ of multi-mutants which were generated by combining multiple stabilizing mutations, purified and *in vitro* characterized for thermal stability by nano-DSF. Details of mutations present in multi-mutants are provided in the **Table 1**. **(I)** mRBD1-3.2 has A348P, Y365W, and P527L mutations. The positions of A348 and Y365 are highlighted in green color. The C-terminal residues (521–526) are highlighted in red color indicating the absence of the electron density of residue 527 in PDB 6M0J. These stabilizing mutations are distant from the receptor-binding motif, highlighted in blue color. **(J)** Neutralizing antibodies can bind to different regions of RBD. The binding epitopes of CR3022, S309, and ACE2 are highlighted by green, red, and blue, respectively.

Earlier studies in other systems have shown that improving thermostability can enhance immunogenicity (29–31). In the present work, we use second-site, saturation suppressor mutagenesis (SSSM) (32, 33) to isolate multiple single-site and multisite stabilized RBD derivatives that were expressed in high yield in mammalian cell culture. The principle of SSSM is as follows. We have previously shown in the context of unstable proteins displayed on the yeast surface that the relative amount of properly folded mutant proteins displayed on the yeast surface correlates with the thermal stability of the corresponding purified mutant measured *in vitro* (34). However, once the stability crosses a certain threshold, further stability increases are not accompanied by increased binding on the yeast surface; hence, it is challenging to isolate mutants with higher stability than the wild type from single-site saturation mutagenesis (SSM) libraries using this approach. To overcome this, a destabilizing mutant (termed as parent inactive mutant or PIM) can be introduced into all members of the mutant library (32, 33) and suppressors isolated (**Figures 1A–D**). A significant fraction of these suppressors is found to be stabilizing even in the wild-type background.

Mouse immunogenicity studies using MF59 equivalent SWE and AddaVax adjuvanted formulations were used to downselect to a single stabilized triple mutant (mRBD1-3.2) which elicited sera that neutralized all four current VOC. Sera from vaccinated as well as convalescent individuals infected during the first wave of the pandemic show a large drop in neutralization titers against the B.1.351 VOC (35, 36). However, sera from individuals infected with B.1.351 neutralize both B.1 and B.1.351 with similar neutralization titers (37). Hence, B.1.351 VOC mutations were introduced into the background of mRBD1-3.2 to give the construct mRBD1-3.2 beta. Since microbial expression is an inexpensive alternative to mammalian cell culture, another derivative named pRBD3-3.2 containing the three stabilizing mutations was also expressed in high yield in the methylotrophic yeast, *P. pastoris.* Like WT monomeric and trimeric RBD described by us previously (28, 38), the stabilized mutants were highly thermotolerant and upon lyophilization were stable at 37°C for over a month. In mice, a cocktail comprising WT and mRBD1-3.2-beta derivatives elicited significant neutralization titers after a single immunization and showed improved breadth relative to mRBD1-3.2. *Pichia*-expressed triple-mutant proteins also elicited broad neutralization of all VOC, although with 1.3–4.6-fold reduced titers relative to mammalian expressed proteins. Hamsters immunized with either mammalian or *Pichia* expressed stabilized RBD were protected from high-dose viral challenge. Overall, the immunogenicity of the stabilized monomers compared favorably with our recently described trimers as well as nanoparticle-displayed RBDs (28, 39–41). In addition, there are no extraneous tag or scaffold-directed antibodies, and permanent cell lines have been made. These data collectively suggest that the present stabilized RBDs are attractive candidates for clinical development and deployment included in remote and low-resource settings.

## Materials and Methods

### Bacterial Strains, Yeast Strains, and Plasmids

*E. coli* strain Top10 was used for cloning and as a host for plasmid propagation. The RBD of SARS-CoV-2 and its mutants were cloned in pCDNA3.4 for expression in Expi293F cells. RBD mutants were cloned in pPICZalphaA for expression in *P. pastoris* X-33 strain (38).

### SSSM Library Generation

The SSSM library was generated as described previously (42). Briefly, an inverse PCR-based approach (43) was used to generate an SSM library of RBD. The RBD gene was cloned in the pUC57 vector, and for a given site, the forward primer had NNK at the 5′ end of the primer and the reverse primer starts at the -1 site, relative to the mutation. In the case of RBD, the residues directly interacting with ACE-2 were not mutated. Individual single-site mutants were generated by inverse PCR, pooled in an equimolar ratio, gel extracted, phosphorylated, and blunt end ligated at 15°C. The ligated product was column purified and transformed in electrocompetent *E. coli* Top10Gyrase cells. Transformed colonies were scraped, and pooled plasmids were purified. The second site suppressor mutant library for RBD was generated by introducing the V512A PIM in the SSM library, as described (32).

### Flow Cytometric Analysis of Yeast Surface Displayed a Single Mutant

Yeast surface display and flow cytometric analysis were performed as described earlier (34). Briefly, *Saccharomyces cerevisiae* EBY100 cells containing WT RBD or mutant plasmids were grown in SDCAA media for 16 h and induced in SGCAA media for 16 h at 30°C. The amount of total protein expressed on the yeast cell surface was estimated by incubating induced cells with chicken anti-myc antibodies (for RBD, 1:400 diluted, from Invitrogen), for 30 min at 4°C. The cells were washed twice and incubated with goat anti-chicken antibodies conjugated to Alexa Flour 488 (1:300 diluted, from Invitrogen), for 20 min at 4°C. The amount of total active protein of RBD on the yeast cell surface was estimated by incubating the induced cells with 100 nM ACE2-hFc for 45 min at 4°C where ACE2-hFc consists of the ACE2 ectodomain fused to human Fc (38). The cells were washed twice and incubated with rabbit anti-human antibodies conjugated to Alexa Fluor 633 (1:1600 diluted, from Invitrogen). Flow cytometric analysis was done on a BD Aria III instrument.

The FACS sample preparation of the RBD SSSM library was performed similar to that for individual mutants of RBD. The number of induced cells and the volume of reagents used were 10-fold higher than for the individually analyzed mutants. Cells were enriched for two rounds to select for suppressor mutants of the PIM which showed improved binding to ACE2. Sanger sequencing of individual colonies of the enriched population was performed, and high-frequency clones were individually analyzed using YSD.

### Expression and Purification of WT and Mutant RBD Proteins

RBD and its mutants were purified as described previously (38) and summarized below. For mammalian cell expression, Expi293F cells were revived and subcultured until the cell density reached approximately 3–5 × 10^6^ cells/ml. On the day of transfection, cells were diluted to a density of 3 × 10^6^ viable cells/ml with pre-warmed media. DNA (1 µg/ml of cell culture) and ExpiFectamine™ 293 reagent were diluted using Opti-MEM™ I medium and incubated at room temperature for 5 min. The diluted DNA and ExpiFectamine™ 293 reagents were mixed and incubated at room temperature for 20 min. The diluted mixture was added to a flask containing cells at a density of 3 × 10^6^ viable cells/ml for transient transfection. Eighteen hours post transfection, ExpiFectamine™ 293 Transfection Enhancer 1 and ExpiFectamine™ 293 Transfection Enhancer 2 were added as per the manufacturer’s instructions. Six days post transfection, the proteins were purified as explained earlier (38), Briefly, the culture supernatant was separated using centrifugation, diluted threefold with PBS, and incubated with Ni Sepharose 6 Fast flow resin for 2 h at 4°C. The unbound fraction was removed, and the column was washed with 50 column volumes of PBS containing 30 mM imidazole. The proteins were eluted using PBS containing 250 mM imidazole, pooled, and dialyzed in PBS. The protein concentration was estimated using NanoDrop™ 2000c with ε_280_ calculated using the ProtParam tool (ExPASy). A polyclonal Flp-In CHO stable line was generated as described previously (28), the mRBD1-3.2 RBD protein expressed at yield of ~60 mg/l.

The RBD mutants were purified from *P. pastoris* as described (38). Briefly, 10 µg pPICZalphaA vector containing the RBD mutant clone, linearized with PmeI enzyme, was transformed in *P. pastoris* X-33 strain using electroporation. Positive transformants were selected on the YPDS plate containing Zeocin (2 mg/ml). Ten colonies were picked and screened for expression. Cells were grown in 8 ml BMMY media (pH 6.0) in a 50-ml shake flask for up to 120 h at 30°C, 250 rpm. The cells were induced with 1% methanol every 24 h, and the expression levels were measured using dot blot analysis with anti-His tag antibodies. The highest expressing colony was chosen for large-scale expression.

Cells were harvested using centrifugation at 4,000g, and the supernatant was passed through a 0.45-µm filter. The supernatant was incubated with pre-equilibrated Ni Sepharose 6 Fast flow resin. The beads were washed with 1× PBS containing 20 mM imidazole and 150 mM NaCl. The protein was eluted using 1× PBS containing 300 mM imidazole and 150 mM NaCl. Fractions containing proteins were pooled and dialyzed against 1× PBS. Tag removal, using HRV3C protease, was carried out as described (38).

### Surface Plasmon Resonance Binding of mRBD1-3.2 and pRBD3-3.2

Protein-binding kinetic studies with ACE2-hFc and CR3022 antibodies were performed on a ProteOn XPR36 Protein Interaction Array V.3.1 (Bio-Rad) as described (38). The GLM sensor chip was activated using EDC (Sigma) and sulfo-NHS. Following activation, protein G (10 µg/ml) was coupled to the chip in 10 mM sodium acetate buffer pH 4.5 at a flow rate of 30 µl/min for 300 s in desired channels to ∼3,500–4,000 RU. Following coupling, unreacted sulfo-NHS ester was quenched using 1 M ethanolamine. Finally, ligands (ACE2-hFc or CR3022) were immobilized at ∼800 RU on desired channels excluding a single blank channel that acts as the reference channel at a flow rate of 30 µl/min. Interaction with ligands was monitored by passing the analyte over the chip at a flow rate of 30 µl/min for 200 s, and the subsequent dissociation phase was monitored for 600 s. An empty lane without ligand immobilization was utilized for measuring non-specific binding. After each kinetic assay, the chip was regenerated with 0.1 M glycine–HCl (pH 2.7). The ligand immobilization cycle was repeated prior to each kinetic assay. Various concentrations of analytes (100, 50, 25, 12.5, 6.25 nM) in 1× PBST were used for binding studies. The kinetic parameters were obtained by fitting the data to a simple 1:1 Langmuir interaction model using ProteOn Manager.

### Long-Term Thermal Stress of mRBD1-3.2

Mammalian cell-expressed WT and mRBD1-3.2 RBD were 1:1 volumetrically diluted in PBS or adjuvants (SWE) at a final concentration of 0.2 mg per ml. The diluted proteins were stored at 4°C and 45°C for up to 28 days. Aliquots were taken at regular intervals and diluted in PBS to a final concentration of 100 nM, and the amount of folded protein remaining was estimated using surface plasmon resonance (SPR).

In another set of experiments, following dialysis against water and lyophilization, RBD was subjected to thermal incubation at 37°C for up to 30 days in individual aliquots. At each time point, aliquots were returned to 4°C. Prior to SPR and differential scanning fluorimetry (DSF), samples were resolubilized in PBS at concentrations of 100 nM and 0.2 mg/ml, respectively. SPR binding to immobilized ACE-2 hFc and DSF were performed as described previously (38).

### Mouse Immunizations

Purified antigen protein (20 µg) was diluted in 50 µl PBS (pH 7.4), mixed with 50 µl of adjuvant (AddaVax™ or SWE) (1:1 v/v antigen: adjuvant), and immunized intramuscularly in groups of five, female, BALB/c mice, (6–8 weeks old, approximately weighing 16–17 g) on Days 0 (prime) and 21 (boost). Blood was collected and serum isolated on days -2 (prebleed), 14, and 35, following the prime and boost immunization, respectively. In a few cases, a second boost was given at day 42. The study was conducted at Central Animal Facility, Indian Institute of Science. All animal studies were approved by the Institutional Animal Ethics committee (IAEC no. CAF/ETHICS/799/2020).

### ELISA-Serum Antibody Endpoint Titers

Serum antibody titers were measured as described (38); briefly, 96-well plates were coated with immunized vaccine antigen, mRBD (38), or spike-2P and incubated for 2 h at 25°C (4 µg/ml, in 1× PBS, 50 µl/well). ACE2-hFc protein coating was used as a control for antigen immobilization. Wells were incubated with blocking solution (100 µl, 3% skimmed milk in 1× PBST) for 1 h at 25°C. Post blocking, antisera (60 µl) starting at 1:100 dilution with fourfold serial dilutions were added, and plates incubated for 1 h at 25°C. Following this, the ALP enzyme conjugated to the rabbit anti-mouse IgG secondary antibody (diluted 1:5,000 in blocking buffer) (50 µl/well) was added, incubated for 1 h at 25°C (Sigma-Aldrich). The wells were washed four times with 1× PBST after each step of immobilization, blocking, and antiserum and secondary antibody binding. pNPP liquid substrate (50 µl/well) (pNPP, Sigma-Aldrich) was added, the plate was incubated for 30 min at 37°C, and the chromogenic signal was measured at 405 nm using an ELISA plate reader (Maxome Labsciences Cat # P3-5x10NO). The highest serum dilution which had a signal twofold above the negative control (empty blocked wells) was considered as the endpoint titer for ELISA.

### Competition ELISA Experiments

Four sets of 96-well plates were coated with mammalian cell-produced WT RBD at 4 µg/ml concentration in 1× PBS (50 µl/well) and incubated for 2 h at 25°C under constant rotation (300 rpm) on a thermomixer (Eppendorf, USA). Ovalbumin (4 µg/ml in 1× PBS, 50 µl/well) coating was used as a negative control for RBD immobilization. Each well was washed with 200 µl/well of 1× PBST followed by blocking solution (100 µl 3% skimmed milk in 1× PBST) for 45 min at 25°C, 300 rpm. Mouse anti-sera were added at twofold serial dilution with a starting dilution of 1:80 in blocking solution (60 µl). Only the blocking solution was added to the control wells. The plates were incubated for 1 h at 25°C, 300 rpm. Three additional washes with 1× PBST were carried out with 200 µl of 1× PBST/well. An additional blocking step was also performed for 45 min with blocking solution (100 µl) incubated at 25°C, 300 rpm. Following this, an excess of either ACE2-hFc, antibody S309, or antibody CR3022 was added (60 µl at 20 µg/ml) to their respective wells and incubated for 1 h at 25°C, 300 rpm. Next, three washes were given (200 µl of PBST/well) to remove excess unbound proteins. Next, 50 µl/well Goat Anti-Human IgG Antibody, alkaline phosphatase conjugate (Sigma-Aldrich Cat # AP112A; diluted 1:5,000 in blocking buffer), was added and samples were incubated for 1 h at 25°C, 300 rpm. Plates were washed four times with 200 µl of PBST/well. Finally, 50 µl/well of a 37°C pre-warmed alkaline phosphatase yellow (pNPP) liquid substrate (Sigma-Aldrich, Cat # P7998) was added, and plates were incubated for 30 min at 37°C, 300 rpm. Finally, the chromogenic signal was measured at 405 nm using an ELISA plate reader (Maxome Labsciences Cat # P3-5x10NO).

The percent competition was calculated using the following equation:

$$\%\text{ Competition}=\frac{\left[\text{Absorbance control}-\text{Absorbance serum dilution}\right]}{\left[\text{Absorbance control}\right]}\times 100$$

where Absorbance control is the absorbance at 405 nm of ACE2-hFc, S309, or CR3022 protein binding directly to mRBD in the absence of sera, and Absorbance serum dilution is the absorbance from wells where the serum dilution is incubated with ACE2-hFc, S309, or CR3022 protein.

The serum dilution factor and % competition data points were fitted using a three-parameter non-linear least-square fit curve using GraphPad Prism 8.4.2. The serum dilution at 50% competition on the fitted curve was termed as the IC_50_ competition titer.

### Convalescent Patient Serum Samples

Convalescent patient sera were drawn (n = 10) and assayed for pseudoviral neutralization as described in the following pseudovirus neutralization section, or for ELISA titers as described above. The ethics approval of human clinical samples was approved by the Institute Human Ethical Committee (Approval No. CSIR-IGIB/IHEC/2020−21/01). Patient informed consent was obtained for obtaining the sera following the Code of the Ethics of the World Medical Association (Declaration of Helsinki).

### Pseudoviral and Replicative Virus Neutralization Assays

Pseudoviral neutralization assays using spike variants displayed on an pHIV-1 NL4.3Δenv-Luc backbone were performed as described (28). Pseudovirus neutralization titers (ID_50_) were determined as the serum dilution at which infectivity was blocked by 50%. Genes encoding spike proteins from VOC were obtained by gene synthesis from GenScript (USA).

Replicative virus culture and neutralization assays were carried out essentially as described previously (28). Briefly, each serum sample was diluted 1:80 in DMEM-D in a deep-well plate, followed by a twofold serial dilution up to 1:163840. The dilution series for each serum sample was dispensed into rows of a 96-well plate, for a total volume of 50 μl per well, and triplicate wells per sample dilution. For the serum-containing wells, 50 μl virus diluted in medium to contain approximately 100 TCID_50_ (checked by back-titration) was added to each well. The plates were incubated at 37°C/5% CO2 for 1 h to allow neutralization complexes to form between the antibodies present in the sera and the virus. At the end of the incubation, 100 μl VeroE6 cells were added to each well and the plates were returned to the incubator for 4 days. Each well was scored for the presence of viral CPE, readily discernible on Day 4 postinfection, with SN_50_ neutralization titers calculated using the Spearman–Karber formula.

### Hamster Experiments

The animal experimental work plans were reviewed and approved by the Indian Institute of Science, Institutional Animals Ethics Committee (IAEC). The experiment was performed according to CPCSEA (The Committee for the Purpose of Control and Supervision of Experiments on Animals) and ARRIVE guidelines. The required number (n = 6/group) of Syrian golden hamsters (*Mesocricetus auratus*) of both sexes (50–60 gm of weight) was procured from the Biogen Laboratory Animal Facility (Bangalore, India). The hamsters were housed and maintained at the Central Animal Facility at IISC, Bangalore, with feed and water ad libitum and a 12-h light and dark cycle. Hamster immunization was performed as described (28). Briefly, animals were acclimatized for 2 weeks and were randomly grouped (n = 6/group). The hamsters were pre-bled and immunized with 20 µg of subunit vaccine candidate in 50 µl injection volume intramuscularly, with the primary on day 0 and boosts on days 21 and 42. Bleeds were performed 2 weeks after each immunization. The hamster immunization study was non-blinded.

Virus challenge was performed as described (28). Briefly, after immunization, the hamsters were transferred to the virus BSL-3 laboratory at the Centre for Infectious Disease Research, Indian Institute of Science-Bangalore (India), and were kept in individually ventilated cages (IVC), maintained at 23 ± 1°C and 50 ± 5% temperature and relative humidity, respectively. After acclimatization of 7 days in IVC cages at the virus BSL-3 laboratory, the hamsters were challenged with 10^6^ PFU of SARS-Cov-2 US strain (USA-WA1/2020 obtained from BEI resources) intranasally in 100 μl of DMEM, by sedating/anaesthetizing the hamsters with a xylazine (10 mg/kg/body wt.) and ketamine (150 g/kg/body wt.) cocktail intraperitoneally. The health of hamsters, body temperatures, body weights, and clinical signs were monitored daily by an expert veterinarian using the scoring system described previously (28). On the fourth day, post challenge, all the hamsters were humanely euthanized by an overdose of xylazine through intraperitoneal injection. The left lobe of the lung was harvested and fixed in 4% paraformaldehyde (PFA) for histopathological examination of lungs. The right lobes were frozen at −80°C for determining the virus copy number by qRT-PCR as described (28).

A histopathological examination of the lung lobes was performed as described (28). Briefly, the left lobes of the lung, fixed in 4% of paraformaldehyde, were processed, embedded in paraffin, and cut into 4-μm sections by a microtome for hematoxylin and eosin staining. The lung sections were microscopically examined and evaluated for different pathological scores by a veterinary immunologist as described (28).

### Statistical Analysis

The p values for ELISA binding and neutralization titers were analyzed with a two-tailed Mann–Whitney test using the GraphPad Prism software 9.0.0 (* indicates p < 0.05, ** indicates p < 0.01, **** indicates p < 0.0001). VOC pseudovirus and replicative virus neutralization titer data were analyzed with non-parametric Kruskal–Wallis with Dunn’s multiple-comparison tests using the GraphPad Prism software 9.0.0 (* indicates p < 0.05, ** indicates p < 0.01, **** indicates p < 0.0001).

## Results

### Second-Site Suppressor Mutagenesis of RBD of SARS-CoV-2

A schematic of the principle of second-site saturation suppressor mutagenesis (SSSM) to isolate stabilizing mutations is shown in **Figures 1A–D**; more detailed descriptions can be found in Sahoo et al. and Ahmed et al. (32, 33). We generated an SSSM library of RBD containing the V512A PIM (parent inactive mutation) and displayed it on the yeast cell surface. Residues involved in ACE2 binding were excluded from mutagenesis. The library was sorted sequentially for two rounds and enriched for improved binders. Individual clones were Sanger sequenced, and flow cytometric analysis of enriched, individual double mutants was performed. The expression mean fluorescence intensity (MFI) of most of the enriched double mutants was similar to that of the PIM (**Figure 1E**) while the binding MFI was higher (**Figure 1F**). Three control mutants with putatively destabilizing mutations at buried sites (Y365V, A410S, V524R) showed lower binding than the PIM. Each of the individual, putatively stabilizing mutations was re-cloned into a mammalian expression vector, expressed, and purified following transient transfection in Expi293 cells as described previously (38). The characterized mutants showed enhancements in thermal stability (T_m_) ranging from 0.7°C to 4.4°C (**Figure 1G**). Upon combining individual stabilizing mutations, we generated several multiple mutants and could enhance T_m_ by 8°C (**Figure 1H**). While combining multiple mutations, we considered only those combinations in which the individual positions have a side-chain centroid–centroid distance of at least 7 Å and are not part of any known neutralizing epitopes. Additional details are provided in **Table 1**. Two of the most stable mutants (mRBD1-3.2, mRBD1-3.3) had yields that were ~30% higher (~160–180 mg/l) than WT. These mutants were stable to incubation for 2 h at 60°C and for up to 20 h at 50°C, showing superior thermal tolerance to the WT (**Supplementary Figure S1**). mRBD1-3.3 had an exposed mutation V367F which is found in some circulating SARS-CoV-2 virus isolates (44). Hence, we primarily focused on detailed characterization of mRBD1-3.2 as it has a Y365W mutation which is buried as well as stabilizing (**Figure 1I**). It also has the A348P and P527L mutations, A348P is also present in SARS-CoV-1, and P527L is located in the disordered C-terminal region of RBD. None of the mutations are present in known neutralizing epitopes or in the receptor-binding motif of RBD (**Figure 1J**). mRBD1 contains two natural N-linked glycosylation sites at residues 331 and 343 and one engineered glycan at residue 532 (38).

**Table 1:** Multi-mutant description.

Multi-mutant	Mutations	RBD start site	RBD end site
mRBD1-2.1	P527L, Y365F	331	532
mRBD1-3.1	P527L, A520G, Y365F	331	532
mRBD1-3.2	A348P, Y365W, P527L	331	532
mRBD1-3.3	A348P, V367F, P527L	331	532
mRBD1-3.5	A348P/V367F/P527I	331	532
mRBD1-4.1	A348P/Y365W/V367F/P527L	331	532
mRBD1-4.2	A348P/Y365W/V367F/P527I	331	532
mRBD1-5.1	T333H, T385S, Y365F, A520G, P527L	331	532
mRBD1-5.2	T333H, A348P, Y365F, A520G, P527L	331	532
mRBD1-6.1	A348P/A372M/P527L/A520G/Y365F/T333H	331	532
mRBD1-7.1	A348P/A372M/T430V/P527L/A520G/Y365F/T333H	331	532
pRBD3-3.2	A348P, Y365W, P527L	332	530
mRBD1-3.2-beta	A348P, Y365W, P527L, K417N, E484K, N501Y	331	532

The first letter denotes expression system used for RBD expression, where m and p correspond to mammalian and Pichia pastoris cells, respectively. The fifth character indicates the start and end sites of RBD, where 1, 2, and 3 correspond to RBD from 331–532, 332–532, and 332–530, respectively. The last two numbers denote the number of mutations in the RBD and a numerical identifier, respectively.

**Figure 2:**
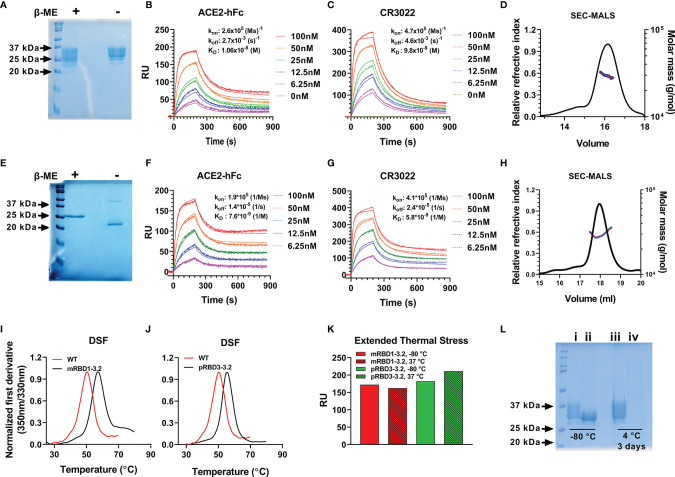
Biophysical characterization of mRBD1-3.2 and pRBD3-3.2. **(A–D)** Characterization of mRBD1-3.2 by **(A)** SDS-PAGE. SPR assays for binding to **(B)** ACE2, **(C)** CR3022, and **(D)** SEC-MALS to estimate molecular mass. **(E–H)** Corresponding data for pRBD3-3.2. Comparison of thermal stability of purified **(I)** mRBD1-3.2 and **(J)** pRBD3-3.2 estimated using nano-DSF. **(K)** Extended thermal stability. Lyophilized mRBD1-3.2 and pRBD3-3.2 were stored at 37°C for 1 month, the lyophilized powder was resolubilized in PBS, and the amount of folded protein was estimated by its binding to ACE-2 hFc using SPR. RU is plotted after 200 s of association with ACE-2. **(L)** Reducing SDS-PAGE analysis of mRBD1-3.2 (i, iii) and mRBD1-3.2-beta (ii, iv) stored at -80°C for over a month and at 4°C for 3 days, respectively.

Our previous attempts (28, 38) at expressing RBD in *P. pastoris* resulted in heterogeneous, hyperglycosylated protein which was poorly immunogenic. Hence, for *P. pastoris* expression we eliminated the glycosylation sites at residues 331 and 532, retained the three stabilizing mutations, and expressed the resulting construct (pRBD3-3.2) with a single glycosylation site at residue 343, in *P. pastoris*. Both mRBD1-3.2 and pRBD3-3.2 had high purified yields of 160–180 and 50–80 mg/l in shake flask cultures of Expi 293F cells and *P. pastoris*, respectively. These proteins were ~100% pure and showed a single protein band for pRBD3-3.2 and multiple bands for mRBD1-3.2 due to variable glycosylation on reducing SDS-PAGE (**Figures 2A, E** **)**. mRBD1-3.2 and pRBD3-3.2 showed binding to soluble ACE2 and CR3022 with affinities similar to WT (**Figures 2B, C, F, G** **)**. Both proteins were monomeric as deduced by SEC-MALS (**Figures 2D, H** **)**. The proteins showed enhanced thermal stability compared to WT (**Figures 2I, J** **)** and retained ACE2 binding when lyophilized and stored at 37°C for over a month (**Figure 2K**). When stored in solution at 4°C for up to 28 days, mRBD1-3.2 retained ACE2 binding both in the presence and in the absence of the MF59 equivalent adjuvant SWE (**Supplementary Figure S2**) and retained significant ACE2 binding even upon storage at 45°C in SWE-adjuvanted solution without additional stabilizing additives for up to 14 days. VOC mutations for B.1.351 were introduced into WT RBD. However, the resultant protein expressed in much lower, 2.5-fold decreased yield, showed lower thermal stability and degraded rapidly when stored at 4°C for 2–3 days (**Supplementary Figure S3A** and **Figure 2L**). However, when the same mutations were introduced in the mRBD1-3.2 background, the protein could be expressed in monomeric form with a high yield of 140 mg/l, showed enhanced thermal stability, and remained stable after purification and extended storage at 4°C (**Supplementary Figures S3A, B**). In a similar fashion, stabilized *Pichia* expressed RBD containing only the B.1.351 mutations degraded rapidly when stored at 4°C, but introduction of the three stabilizing mutations into this background resulted in protein that was stable for extended periods at 4°C.

### Mouse Immunizations

**Figure 3:**
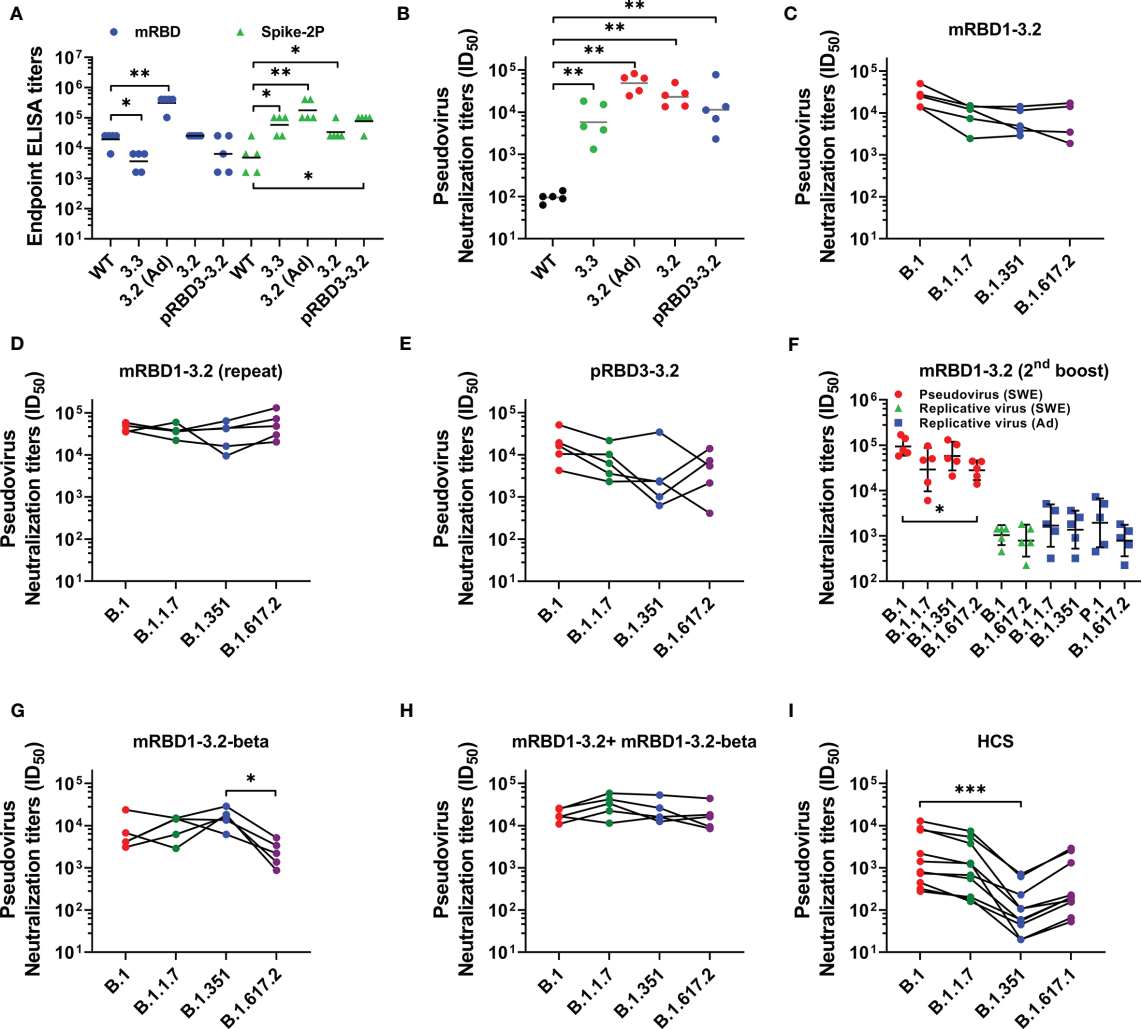
Comparative immunogenicity data. Mice were immunized on prime (day 0) and boost (day 21) except for the data in **(F)** which are after a third immunization at day 42. Unless otherwise specified, proteins were formulated with SWE. **(A)** ELISA endpoint titers elicited by WT and mutant RBDs against mRBD and Spike-2P ectodomain. **(B)** Neutralizing antibody titers elicited with WT and mutant RBDs against WT (B.1) pseudovirus. Neutralizing antibodies titer against WT, and VOC pseudoviruses were elicited after two immunizations with **(C, D)** mRBD1-3.2 and **(E)** pRBD3-3.2. **(F)** Pseudoviral and replicative virus neutralization titers after three immunizations spaced 3 weeks apart, using mRBD1-3.2 formulated with either SWE or AddaVax. The pseudoviral data for the SWE formulation after two immunizations are shown in **(D)**. Pseudoviral neutralization titers elicited after two immunizations by **(G)** mRBD1-3.2-beta and **(H)** cocktail formulation of mRBD1-3.2 and mRBD1-3.2-beta. **(I)** Pseudoviral neutralization titers of HCS. p values for comparison of ELISA binding titers and neutralization titers were calculated with a two-tailed Mann–Whitney test. The paired comparisons of pseudoviral and replicating virus neutralization titers were performed utilizing the Kruskal–Wallis with Dunn’s multiple-comparison tests. A significant difference was only seen for **(F)** (between B.1 and B.1.617.2 pseudoviruses, p = 0.0163), **(G)** (between B.1.351 and B.1.617.2 pseudoviruses, p = 0.0116), and **(I)** (between B.1 and B.1.351, p = 0.0007). *, **, and *** indicate p < 0.05, <0.01, and < 0.001, respectively.

The immunogens were formulated in either AddaVax or SWE, which are oil-in-water emulsions that are both near identical to each other and to the MF59 adjuvant. The formulations were used to immunize mice at day 0 (prime) and day 21 (boost). Sera were collected 2 weeks after each immunization. We have previously shown that both adjuvants elicit similar antibody titers (28). Post boost, mice immunized with the stabilized mutants showed high geometric mean titers (GMT) against Spike S-2P as well as against RBD (**Figure 3A**). Relative to RBD lacking the stabilizing mutations, the stabilized mutants elicited considerably higher binding and neutralizing antibody titers against the B.1 pseudovirus (**Figures 3A, B** **)**. Sera elicited by the SWE-adjuvanted mRBD1-3.2 also neutralized SARS-CoV-2 VOC pseudoviruses (**Figure 3C**). To further validate this result, an independent biological repeat immunization with mRBD1-3.2 was carried out. The results for the repeat studies are shown in **Figure 3D**. The neutralization data are similar for both the original and repeat studies, although the neutralization titers in the repeat study are qualitatively higher and less sensitive to VOC mutations than those reported in the first study. The *Pichia*-expressed stabilized mutant also showed neutralization of B.1 and VOC, although with slightly lower titers than mammalian-expressed proteins (**Figure 3E**). mRBD1-3.2 elicited sera from two independent studies were also tested for neutralization of pseudovirus and replicative virus (**Figure 3F**). The sera used in **Figure 3F** were after three immunizations, since the sera after two immunizations were largely used up in the pseudoviral assays (**Figure 3D**). The pseudoviral titers after two and three immunizations are similar (compare **Figures 3D, F** **)**. The neutralization titers were largely insensitive to VOC mutations (**Figure 3F**).

SWE-adjuvanted mRBD1-3.2-beta as well as a cocktail of mRBD1-3.2+ mRBD1-3.2-beta elicited high titers of antibodies (**Supplementary Figure 3C**). The cocktail displayed more uniform neutralization of VOC than mRBD1-3.2 or mRBD1-3.2-beta alone (**Figures 3C, G, H** **)**. Using the present pseudoviral assay, the GMT neutralization titer observed for a set of human convalescent sera (HCS) against B.1 was ~137 (28). Hence, in order to measure the fold change in neutralization titer against VOC, a subset of high titer HCS was used as lower titer sera lost neutralization against B.1.351 (**Figure 3I**). It is known from other studies (28) that in human sera/plasma from both convalescent and vaccinated individuals, there is a significant drop in neutralization titers against VOC, in particular B.1.351, relative to those observed against B.1. We too observe similar results (**Figure 3I**), thus validating the pseudoviral constructs that we had made. Therefore, in the animal immunizations reported in the present study, the relative lack of sensitivity of elicited antibodies to VOC is significant (**Figure 3**). To map epitopes targeted in the various sera, we carried out competition binding of sera with mRBD1-3.2-binding mAbs (CR3022 and S309) as well as with ACE2-hFc to RBD (**Supplementary Figure S4**). We found significant competition of sera only with ACE2-hFc. We also measured ELISA titers of HCS against the WT and stabilized RBDs (mRBD1-3.2 and pRBD3-3.2) as well as the Spike-2P ectodomain (3, 45). ELISA titers for binding WT and stabilized RBDs were both comparable to those for the Spike-2P protein (**Supplementary Figure S5**).

### Hamster Immunization and Challenge Studies

**Figure 4:**
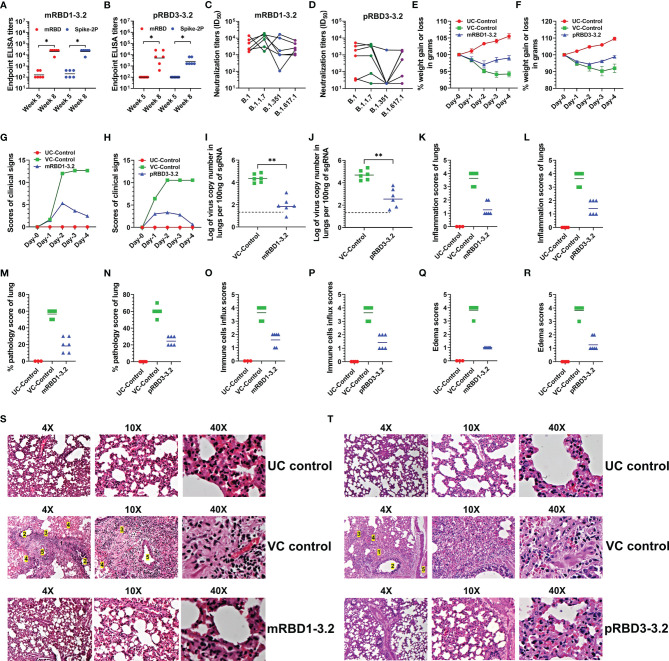
Comparative immunogenicity and challenge data in hamsters. Hamsters immunized with mRBD1-3.2 or pRBD3-3.2 adjuvanted with SWE on weeks 0, 3, and 6. Animals were bled 2 weeks after each immunization. Comparison of IgG titers against mRBD and Spike-2P elicited by **(A)** mRBD1-3.2 and **(B)** pRBD3-3.2. Neutralization titers against pseudoviruses containing VOC RBD mutations elicited by **(C)** mRBD1-3.2 and **(D)** pRBD3-3.2. After three immunizations, hamsters were challenged intranasally with replicative SARS-CoV-2 virus (10^6^ pfu/hamster). The hamsters were monitored for **(E, F)** weight change, **(G, H)** clinical signs, and **(I, J)** virus copy number in lungs. Histopathology score of lungs including **(K, L)** inflammation, **(M, N)** pathology, **(O, P)** immune cells influx, and **(Q, R)** edema score. Histology of lung sections at varying magnifications (×4, ×10, and ×40). Lung pathologies of unchallenged control (UC), unimmunized and virus challenged (VC) animals, and animals immunized with **(S)** mRBD1-3.2 or **(T)** pRBD3-3.2. Unchallenged animals showed normal lung parenchyma with minimal alveolar inflammation. Lung histology images were marked to identify 1) severe congestion of alveolar space, alveolar inflammation, alveolar edema, and diffuse alveolar damage; 2) bronchial inflammation; 3) infiltration of immune cells, leukocytic alveolitis, and necrosis; 4) blood hemorrhage, and 5) perivascular inflammation, edema, and vascular congestion. Immunized hamsters showed normal lung parenchyma with minimum to moderate alveolar and bronchial inflammation. The p values for comparison of ELISA binding titers; virus copy numbers in lungs were computed with a two-tailed Mann–Whitney test. The paired comparisons of pseudoviral neutralization titers were performed utilizing the Kruskal–Wallis test with Dunn’s multiple-comparison test. p value indicated with * and ** indicates < 0.05 and < 0.01, respectively.

We next examined the efficacy of mRBD1-3.2 and pRBD3-3.2 respectively in two independent hamster immunization and challenge studies. Hamsters were immunized with SWE-adjuvanted formulations at weeks 0, 3, and 6 and subjected to a high-dose intranasal challenge with 10^6^ pfu of replicative SARS-CoV-2 virus (USA-WA1/2020). ELISA titers are shown in **Figures 4A, B** and pseudoviral neutralizing titers in **Figures 4C, D**. Overall, the neutralizing titers elicited with *Pichia*-produced proteins were lower and more sensitive to the mutations in the B.1.351 VOC than corresponding sera elicited by mammalian cell-expressed proteins.

Following immunization, the animals were challenged with replicative WT SARS-CoV-2 virus. We also included two additional groups as controls, namely, unimmunized-unchallenged (UC) and unimmunized-virus challenged (VC) animals. Post infection, vaccinated animals showed a slight weight reduction but regained weight after 3 days, in contrast to the VC control group (**Figures 4E, F** **)**. The immunized hamsters also showed lower clinical signs (**Figures 4G, H** **)**, lung viral titers (**Figures 4I, J** **)**, and histopathology scores (**Figures 4K–T**) than the VC control group. The tissue sections show minimal immune cell infiltration and clear lung epithelial interstitial spaces in the immunized group compared to the VC control group.

In summary, both mRBD1-3.2 and pRBD3-3.2 elicited robust binding and neutralizing antibodies which protected hamsters from high-dose replicative viral challenge. Future, additional preclinical studies will assess the immunogenicity and protective efficacy of stabilized RBD in transgenic mice expressing human Ace2 to further assess the utility of this antigen in a vaccine.

## Discussion

SARS-CoV-2 is the etiological agent of the ongoing pandemic which has so far resulted in over four million deaths worldwide. Multiple vaccines have been approved for emergency use. However, there remains a need for a cheap, highly scalable, efficacious vaccine which elicits antibodies that neutralize VOC and preferably does not require a cold chain. It has previously been shown in the context of HIV-1 antigens that stabilized mutants of a protein can enhance immunogenicity (29–31, 46). We recently described a workflow employing second-site saturation-suppressor mutagenesis (SSSM) coupled to yeast surface display to rapidly identify stabilizing mutations (33). Recent work describes related approaches to identify stabilizing point mutants of GFP and CI2 from multi-mutant libraries in *E. coli* (47–49). In the present study, we employ this to identify stabilizing mutations of the receptor-binding domain (RBD) of the spike protein of SARS-CoV-2. We identified numerous individual stabilization mutations which enhance T_m_ by 0.7°C to 4.4°C. We combined multiple stabilizing mutations resulting in a triple mutant with an increase in T_m_ of ~8°C. Incorporation of additional stabilization mutations did not further enhance the thermal stability. Two stabilized multi-mutants, namely, mRBD1-3.2 and mRBD1-3.3, were selected for additional characterization and showed 30% higher yield compared to the corresponding WT.

In our previous work, we found that *Pichia*-expressed RBD immunogens were more heterogeneous than their mammalian counterparts and failed to elicit neutralizing antibodies after two immunizations in mice or guinea pigs (28, 38). However, those earlier immunogens were based on WT sequence. Since *Pichia* offers a low-cost, high-volume microbial alternative to mammalian cell culture, in the present work we explored if *Pichia* RBD with stabilizing mutations performed better than the corresponding WT as well as mammalian cell-expressed proteins with the same stabilizing mutations. A construct containing the stabilizing mutations present in mRBD1-3.2 was therefore expressed in *P. pastoris*. Both mammalian and *Pichia*-expressed proteins were homogenous and monomeric and bound tightly to the conformation specific ligands, ACE-2 hFc and CR3022. The proteins also remained folded when lyophilized from either water or PBS. The lyophilized protein remained stable when stored at 37°C for over a month. Proteins in PBS buffer also remained folded when stored at 4°C for at least 28 days and 45°C for up to 7–14 days in the presence and absence of adjuvant SWE in solution. Our previous attempts at expressing monomeric or trimeric RBD in *Pichia* (28, 38) yielded heterogeneous, highly glycosylated proteins, which when formulated with SWE was poorly immunogenic in mice and elicited very low titers of neutralizing antibodies. In contrast, in the present study the stabilized pRBD3-3.2 was very homogenous. The stabilized mutants mRBD1-3.2 and pRBD3-3.2 elicited neutralizing antibodies against VOC albeit with the *Pichia*-expressed protein eliciting lower neutralization titers than mammalian-expressed protein (**Figure 3E**).

It has been previously observed (35–37) that while convalescent sera from B.1-infected individuals poorly neutralized B.1.351, convalescent sera from B.1.351-infected individuals neutralized both B.1 and B.1.351, suggesting that immunogens based on B.1.351 sequence might provide increased breadth of protection against VOC. However, this does not appear to be the case. Mice immunized with mRBD1-3.2-beta alone showed a significant decrease in titers for B.1.617.2 VOC, while this was not the case for mice immunized with mRBD1-3.2 (compare **Figures 3D, G** **)**. It will be interesting and important to see if humans infected with B.1.351 show a similar trend of reduced neutralization and increased susceptibility to B.1.617.2. A cocktail of the above immunogens showed uniformly high neutralization titers against the various VOC (**Figure 3H**). These data suggest that vaccines based on either B.1 (ancestral) or an appropriate cocktail are likely to offer the best protection against current and closely related VOC.

Hamsters immunized with either mRBD1-3.2 or pRBD3-3.2 were well protected against high-dose viral challenge. Immunized animals showed substantial improvement in clinical signs, lung viral titers, and histopathology compared to unimmunized hamsters challenged with the virus. mRBD1-3.2-immunized hamsters showed improved neutralization breadth relative to pRBD3-3.2 immunized animals. There are a few differences between the *Pichia*- and mammalian cell-expressed proteins. Firstly, *Pichia*-produced proteins have high mannose glycans unlike mammalian cell-expressed proteins which can have complex glycans including terminal sialic acid residues. It is known from other studies that the chemical composition of incorporated glycans can alter immunogenicity (50, 51) as well as antigen half-life (52). Secondly, the *Pichia*-expressed protein pRBD3-3.2 has a single N-linked glycosylation site at residue 343. In contrast, the mammalian-expressed mRBD1-3.2 protein has two additional glycosylation sites at residues 331 and 532 in addition to the one at residue 343. We did not include the additional sites in the *Pichia*-expressed molecule because our prior experience with RBD expression in *Pichia* (28, 38), suggesting that incorporation of additional glycosylation sites led to formation of heterogeneous, hyperglycosylated molecules. It is thus possible that the altered glycosylation present in mammalian cell-expressed proteins as well as the presence of two additional glycosylation sites at the N and C termini improves the immunogenicity of mammalian cell-expressed mRBD1-3.2 relative to the *Pichia*-expressed protein.

*P. pastoris*-expressed pRBD3-3.2 provides an inexpensive platform for the large-scale production of affordable vaccines. *Pichia* expression technology is widely available in several developing countries including India, Bangladesh, Brazil, Cuba, and Indonesia (53). There have been other attempts to express RBD in *Pichia*, both as a monomeric protein (53–55) and displayed on the surface of hepatitis B VLPs (56); both are in clinical trials, but data from the trials are not currently available. In the case of the hepatitis B VLP-RBD, neutralization titers against B.1.1.7 were comparable to those against B.1; however, titers against the B.1.351 titer were ~25-fold lower than against B.1 (56). Recently, a deep mutational scanning-combined structure-guided mutational prediction approach led to generation of an RBD multi-mutant Rpk9 that showed enhanced thermostability (ΔT_m_: 3.8°C) and yield (41). In the context of a self-assembling two component nanoparticle, the above mutant showed similar immunogenicity to the corresponding WT derivative but increased thermotolerance in storage buffer containing glycerol and arginine. The Y365F, V395I, and F392W mutations present in Rpk9 are in the vicinity of a linoleic binding cavity, different from the ones in the present study, although one site, Y365, is common. In another study, a *Pichia*-expressed RBD (residues 332-549, C538A) adjuvanted with 3M-052 + alum, when used in macaque immunizations, elicited neutralizing antibodies that showed an ~5.4-fold decrease in ID_50_ against B.1.351, relative to B.1 in a pseudoviral neutralization assay (55). It is difficult to directly compare animal immunogenicity data since different studies have used different animal models, adjuvants, and neutralization assays. However, it is likely that the stabilizing mutations we have identified could be used to improve yield, immunogenicity, and stability of the above RBD derivatives as well. Monomeric RBD is likely easier to manufacture and characterize than VLP or self-assembling nanoparticle displayed material (39, 56–60), and there are no extraneous responses produced against the other components of the VLP which can be quite immunogenic (61–63). The improvement in immunogenicity with multivalent display is most apparent after a single immunization (41). The current *Pichia*-expressed, stabilized RBD shows considerably enhanced immunogenicity relative to a similar construct without the stabilizing mutations which we described previously (38), and an AddaVax-adjuvanted formulation of the latter construct failed to elicit neutralizing antibodies (38). Relative to our recently described trimeric RBD (28), the present stabilized monomer shows comparable to superior immunogenicity without the possible complication of trimerization scaffold-directed antibodies. The stabilizing mutations can also be incorporated into RBDs from VOC. In the present case, we showed that they considerably enhanced expression yield and stability of RBD derived from the B.1.351 VOC. While the stabilized mRBD1-3.2 RBD derived from the B.1 sequence elicited antibodies which neutralized all VOC, inclusion of mRBD1-3.2-beta RBD into the formulation further increased the neutralization titers and neutralization breadth relative to antisera elicited by either component alone. However, whether this lack of sensitivity to VOC also holds true in humans immunized with the same formulation can only be ascertained after clinical trials. In summary, we describe the isolation of a high-yielding, thermostable, and thermotolerant monomeric RBD vaccine candidate. In mice and hamsters, this formulation elicits antibodies that neutralize all current VOC and is currently being moved forward to clinical development. In addition, the work demonstrates that saturation suppressor methodology is a facile approach to stabilize proteins for vaccine and therapeutic application.

## Data Availability Statement

The original contributions presented in the study are included in the article/**Supplementary Material**. Further inquiries can be directed to the corresponding authors.

## Ethics Statement

The studies involving human participants were reviewed and approved by the Institute Human Ethical Committee of CSIR-IGIB (Approval No. CSIR-IGIB/IHEC/2020−21/01). The patients/participants provided their written informed consent to participate in this study. The animal study was reviewed and approved by Institutional Animal Ethics Committee of Indian Institute of Science (IAEC no. CAF/ETHICS/799/2020).

## Author Contributions

RV and SA conceptualized the work and designed the studies. SA performed all YSD studies, purified mammalian cell-expressed RBD mutants, and characterized them by DSF. MK expressed the RBD mutants in mammalian cells, assisted with some of the purifications, and performed SEC-MALS and some of the DSF studies. SGa cloned, expressed, and characterized the *Pichia* RBD derivatives. SP, RV, and RRa planned the animal studies. RS constructed RBDs with VOC mutations and stable CHO lines and performed monoclonal antibody and ACE2 competition with immunized mouse sera. RS, UP, PR, and AU performed ELISA. OK, RN, and ST standardized the growth and quantitation of replicative virus. RRa performed the hamster immunizations and challenge studies. UP, SP, NG, and AU performed the protein expression and characterization of VOC RBDs. SKM carried out the DSF and SPR characterization. RRi and SKu designed, optimized, and executed the pseudoviral neutralization assays and analyzed the data. RRi was also involved across all the stages of the work. PvV, SR, SGo, SS, AT, SJ, and RP provided convalescent human serum samples. SV and RV discussed the development of mRBD1-3.2+ mRBD1-3.2-beta cocktail. PvV, SR, SGo, AM, NS, and SV designed, optimized, and executed the live virus neutralization assays and analyzed the data. SA and RV wrote the manuscript with contributions from each author. All authors contributed to the article and approved the submitted version.

## Funding

This work was funded by a grant from the Bill and Melinda Gates Foundation (INV-005948) and from the Office of the Principal Scientific Advisor, Government of India (SP/OPSA-20-0004) to RV, and by a major grant from the Australia’s Department of Finance to CSIRO (P.I.: SV). We also acknowledge funding for infrastructural support from the following programs of the Government of India: DST-FIST, UGC Center for Advanced Study, MHRD-FAST, and the DBT-IISc Partnership Program, and of a JC Bose Fellowship from DST to RV. Mynvax acknowledges funding support from IISc CSR grant for COVID-19 vaccine work. RP acknowledges the support of CSIR-IGIB grant (MLP-2005) and Fondation Botnar (CLP-0031). RRi acknowledges the support of the SERB grant (IPA/2020/000168). ST acknowledges funding support from DBT-BIRAC (BT/CS0007/CS/02/20) and DBT-WT India Alliance (IA/I/18/1/503613). The content is solely the responsibility of the authors and does not necessarily represent the official views of the funding institutions.

## Conflict of Interest

A provisional patent application has been filed for the RBD formulations described in this article. SKM, SA, RV, SP, and RS are inventors. RV is founder of Mynvax, and SP, RS, NG, AU, and PR are employees of Mynvax Private Limited.

The remaining authors declare that the research was conducted in the absence of any commercial or financial relationships that could be construed as a potential conflict of interest.

## Publisher’s Note

All claims expressed in this article are solely those of the authors and do not necessarily represent those of their affiliated organizations, or those of the publisher, the editors and the reviewers. Any product that may be evaluated in this article, or claim that may be made by its manufacturer, is not guaranteed or endorsed by the publisher.

## References

[1] WHO Covid-19. WHO Coronavirus Disease (COVID-19) Dashboard, in: World Heal Organ (2020). Available at: https://covid19.who.int/ (Accessed August 27, 2020).

[2] Huang Y Sun H Yu H Li S Zheng Q Xia N. Neutralizing Antibodies Against SARS-CoV-2: Current Understanding, Challenge and Perspective. Antib Ther (2020) 3:285–99. doi: 10.1093/abt/tbaa028 PMC7799234 33912797

[3] Wrapp D Wang N Corbett KS Goldsmith JA Hsieh C-L Abiona O. Cryo-EM Structure of the 2019-Ncov Spike in the Prefusion Conformation. Science (2020) 367:1260–3. doi: 10.1126/science.abb2507 PMC7164637 32075877

[4] Shang J Wan Y Luo C Ye G Geng Q Auerbach A. Cell Entry Mechanisms of SARS-CoV-2. Proc Natl Acad Sci USA (2020) 117:11727–34. doi: 10.1073/pnas.2003138117 PMC7260975 32376634

[5] Piccoli L Park YJ Tortorici MA Czudnochowski N Walls AC Beltramello M. Mapping Neutralizing and Immunodominant Sites on the SARS-CoV-2 Spike Receptor-Binding Domain by Structure-Guided High-Resolution Serology. Cell (2020) 183:1024–42.e21. doi: 10.1016/j.cell.2020.09.037 32991844 PMC7494283

[6] Liu L Wang P Nair MS Yu J Rapp M Wang Q. Potent Neutralizing Antibodies Against Multiple Epitopes on SARS-CoV-2 Spike. Nature (2020) 584:450–6. doi: 10.1038/s41586-020-2571-7 32698192

[7] Zost SJ Gilchuk P Chen RE Case JB Reidy JX Trivette A. Rapid Isolation and Profiling of a Diverse Panel of Human Monoclonal Antibodies Targeting the SARS-CoV-2 Spike Protein. Nat Med (2020) 26:1422–7. doi: 10.1038/s41591-020-0998-x PMC8194108 32651581

[8] van Doremalen N Lambe T Spencer A Belij-Rammerstorfer S Purushotham JN Port JR. ChAdOx1 Ncov-19 Vaccine Prevents SARS-CoV-2 Pneumonia in Rhesus Macaques. Nature (2020) 586:578–82. doi: 10.1038/s41586-020-2608-y PMC8436420 32731258

[9] Zhu FC Guan XH Li YH Huang JY Jiang T Hou LH. Immunogenicity and Safety of a Recombinant Adenovirus Type-5-Vectored COVID-19 Vaccine in Healthy Adults Aged 18 Years or Older: A Randomised, Double-Blind, Placebo-Controlled, Phase 2 Trial. Lancet (2020) 396:479–88. doi: 10.1016/S0140-6736(20)31605-6 PMC7836858 32702299

[10] Logunov DY Dolzhikova IV Shcheblyakov DV Tukhvatulin AI Zubkova OV Dzharullaeva AS. Safety and Efficacy of an Rad26 and Rad5 Vector-Based Heterologous Prime-Boost COVID-19 Vaccine: An Interim Analysis of a Randomised Controlled Phase 3 Trial in Russia. Lancet (2021) 397:671–81. doi: 10.1016/S0140-6736(21)00234-8 PMC7852454 33545094

[11] Sadoff J Le Gars M Shukarev G Heerwegh D Truyers C de Groot AM. Safety and Immunogenicity of the Ad26.COV2.S COVID-19 Vaccine Candidate: Interim Results of a Phase 1/2a, Double-Blind, Randomized, Placebo-Controlled Trial. medRxiv (2020) 1–28. doi: 10.1101/2020.09.23.20199604

[12] Marsh GA McAuley AJ Au GG Riddell S Layton D Singanallur NB. ChAdOx1 Ncov-19 (AZD1222) Vaccine Candidate Significantly Reduces SARS-CoV-2 Shedding in Ferrets. NPJ Vaccines 2021 61 (2021) 6:1–8. doi: 10.1038/s41541-021-00315-6 PMC8110954 33972565

[13] Liu R Americo JL Cotter CA Earl PL Erez N Peng C. One or Two Injections of MVA-Vectored Vaccine Shields Hace2 Transgenic Mice From SARS-CoV-2 Upper and Lower Respiratory Tract Infection. Proc Natl Acad Sci USA (2021) 118:e2026785118. doi: 10.1073/pnas.2026785118 33688035 PMC8000198

[14] Routhu NK Cheedarla N Gangadhara S Bollimpelli VS Boddapati AK Shiferaw A. A Modified Vaccinia Ankara Vector-Based Vaccine Protects Macaques From SARS-CoV-2 Infection, Immune Pathology, and Dysfunction in the Lungs. Immunity (2021) 54:542–56.e9. doi: 10.1016/j.immuni.2021.02.001 33631118 PMC7859620

[15] Mulligan MJ Lyke KE Kitchin N Absalon J Gurtman A Lockhart S. Phase I/II Study of COVID-19 RNA Vaccine BNT162b1 in Adults. Nature (2020) 586:589–93. doi: 10.1038/s41586-020-2639-4 32785213

[16] Baden LR El Sahly HM Essink B Kotloff K Frey S Novak R. Efficacy and Safety of the mRNA-1273 SARS-CoV-2 Vaccine. N Engl J Med (2021) 384:403–16. doi: 10.1056/nejmoa2035389 PMC7787219 33378609

[17] Riddell S Goldie S McAuley AJ Kuiper MJ Durr PA Blasdell KR. Live Virus Neutralisation of the 501Y.V1 and 501Y.V2 SARS-CoV-2 Variants Following INO-4800 Vaccination of Ferrets. Front Immunol (2021) 0:694857. doi: 10.3389/FIMMU.2021.694857 PMC8269317 34248993

[18] Ella R Reddy S Jogdand H Sarangi V Ganneru B Prasad S. Safety and Immunogenicity of an Inactivated SARS-CoV-2 Vaccine, BBV152: Interim Results From a Double-Blind, Randomised, Multicentre, Phase 2 Trial, and 3-Month Follow-Up of a Double-Blind, Randomised Phase 1 Trial. Lancet Infect Dis (2021) 21:950–61. doi: 10.1016/s1473-3099(21)00070-0 PMC8221739 33705727

[19] Gao Q Bao L Mao H Wang L Xu K Yang M. Development of an Inactivated Vaccine Candidate for SARS-CoV-2. Science (2020) 369:77–81. doi: 10.1126/science.abc1932 32376603 PMC7202686

[20] Tian JH Patel N Haupt R Zhou H Weston S Hammond H. SARS-CoV-2 Spike Glycoprotein Vaccine Candidate NVX-CoV2373 Immunogenicity in Baboons and Protection in Mice. Nat Commun (2021) 12:1–14. doi: 10.1038/s41467-020-20653-8 33446655 PMC7809486

[21] WHO Covid-19. Draft Landscape of COVID-19 Candidate Vaccines (2020). Who. Available at: https://www.who.int/publications/m/item/draft-landscape-of-covid-19-candidate-vaccines (Accessed September 18, 2020).

[22] Wibmer CK Ayres F Hermanus T Madzivhandila M Kgagudi P Oosthuysen B. SARS-CoV-2 501y.V2 Escapes Neutralization by South African COVID-19 Donor Plasma. Nat Med (2021) 27:622–5. doi: 10.1038/s41591-021-01285-x 33654292

[23] Baum A Fulton BO Wloga E Copin R Pascal KE Russo V. Antibody Cocktail to SARS-CoV-2 Spike Protein Prevents Rapid Mutational Escape Seen With Individual Antibodies. Science (2020) 369:1014–8. doi: 10.1126/science.abd0831 PMC7299283 32540904

[24] Chen RE Zhang X Case JB Winkler ES Liu Y VanBlargan LA. Resistance of SARS-CoV-2 Variants to Neutralization by Monoclonal and Serum-Derived Polyclonal Antibodies. Nat Med (2021) 27:717–26. doi: 10.1038/s41591-021-01294-w PMC8058618 33664494

[25] Kupferschmidt K Wadman M. Delta Variant Triggers New Phase in the Pandemic. Science (2021) 372:1375–6. doi: 10.1126/science.372.6549.1375

[26] Alizon S Haim-Boukobza S Foulongne V Verdurme L Trombert-Paolantoni S Lecorche E. Rapid Spread of the SARS-CoV-2 Delta Variant in Some French Regions, June 2021. Eurosurveillance (2021) 26:2100573. doi: 10.2807/1560-7917.ES.2021.26.28.2100573 PMC8284044 34269174

[27] O’Dowd A. Covid-19: Cases of Delta Variant Rise by 79%, But Rate of Growth Slows. BMJ (2021) 373:n1596. doi: 10.1136/BMJ.N1596 34154997

[28] Malladi SK Patel UR Rajmani RS Singh R Pandey S Kumar S. Immunogenicity and Protective Efficacy of a Highly Thermotolerant, Trimeric SARS-CoV-2 Receptor Binding Domain Derivative. ACS Infect Dis (2021) 7:2546–64. doi: 10.1021/acsinfecdis.1c00276 PMC8996237 34260218

[29] Torrents de la Peña A Julien JP de Taeye SW Garces F Guttman M Ozorowski G. Improving the Immunogenicity of Native-Like HIV-1 Envelope Trimers by Hyperstabilization. Cell Rep (2017) 20:1805–17. doi: 10.1016/j.celrep.2017.07.077 PMC5590011 28834745

[30] De Taeye SW Ozorowski G Torrents de la Peña A Guttman M Julien JP Van Den Kerkhof TLGM. Immunogenicity of Stabilized HIV-1 Envelope Trimers With Reduced Exposure of Non-Neutralizing Epitopes. Cell (2015) 163:1702–15. doi: 10.1016/j.cell.2015.11.056 PMC4732737 26687358

[31] Feng Y Tran K Bale S Kumar S Guenaga J Wilson R. Thermostability of Well-Ordered HIV Spikes Correlates With the Elicitation of Autologous Tier 2 Neutralizing Antibodies. PloS Pathog (2016) 12:e1005767. doi: 10.1371/JOURNAL.PPAT.1005767 27487086 PMC4972253

[32] Sahoo A Khare S Devanarayanan S Jain PC Varadarajan R. Residue Proximity Information and Protein Model Discrimination Using Saturation-Suppressor Mutagenesis. Elife (2015) 4:e09532. doi: 10.7554/eLife.09532 26716404 PMC4758949

[33] Ahmed S Manjunath K Varadarajan R. Stabilizing Proteins Through Saturation Suppressor Mutagenesis. bioRxiv (2021) 2021.08.07.455542. doi: 10.1101/2021.08.07.455542

[34] Ahmed S Manjunath K Varadarajan R. Prediction of Residue-Specific Contributions to Binding and Thermal Stability Using Yeast Surface Display. bioRxiv (2021) 2021.05.31.446445. doi: 10.1101/2021.05.31.446445 PMC8814602 35127820

[35] Zhou D Dejnirattisai W Supasa P Liu C Mentzer AJ Ginn HM. Evidence of Escape of SARS-CoV-2 Variant B.1.351 From Natural and Vaccine-Induced Sera. Cell (2021) 184:2348–61.e6. doi: 10.1016/J.CELL.2021.02.037 33730597 PMC7901269

[36] Garcia-Beltran WF Lam EC St Denis K Nitido AD Garcia ZH Hauser BM. Multiple SARS-CoV-2 Variants Escape Neutralization by Vaccine-Induced Humoral Immunity. medRxiv (2021). doi: 10.1101/2021.02.14.21251704 PMC8082941 33930298

[37] Moyo-Gwete T Madzivhandila M Makhado Z Ayres F Mhlanga D Oosthuysen B. Cross-Reactive Neutralizing Antibody Responses Elicited by SARS-CoV-2 501y.V2 (B.1.351). N Engl J Med (2021) 384:2161–3. doi: 10.1056/NEJMC2104192 PMC8063886 33826816

[38] Malladi SK Singh R Pandey S Gayathri S Kanjo K Ahmed S. Design of a Highly Thermotolerant, Immunogenic SARS-CoV-2 Spike Fragment. J Biol Chem (2021) 296:100025. doi: 10.1074/jbc.RA120.016284 33154165 PMC7832000

[39] Tan TK Rijal P Rahikainen R Keeble AH Schimanski L Hussain S. A COVID-19 Vaccine Candidate Using SpyCatcher Multimerization of the SARS-CoV-2 Spike Protein Receptor-Binding Domain Induces Potent Neutralising Antibody Responses. Nat Commun (2021) 12:1–16. doi: 10.1038/s41467-020-20654-7 33483491 PMC7822889

[40] Routhu NK Cheedarla N Bollimpelli VS Gangadhara S Edara VV Lai L. SARS-CoV-2 RBD Trimer Protein Adjuvanted With Alum-3M-052 Protects From SARS-CoV-2 Infection and Immune Pathology in the Lung. Nat Commun (2021) 12:3587. doi: 10.1038/s41467-021-23942-y 34117252 PMC8196016

[41] Ellis D Brunette N Crawford KHD Walls AC Pham MN Chen C. Stabilization of the SARS-CoV-2 Spike Receptor-Binding Domain Using Deep Mutational Scanning and Structure-Based Design. Front Immunol (2021) 12:710263. doi: 10.3389/fimmu.2021.710263 34267764 PMC8276696

[42] Adkar BV Tripathi A Sahoo A Bajaj K Goswami D Chakrabarti P. Protein Model Discrimination Using Mutational Sensitivity Derived From Deep Sequencing. Structure (2012) 20:371–81. doi: 10.1016/j.str.2011.11.021 22325784

[43] Jain PC Varadarajan R. A Rapid, Efficient, and Economical Inverse Polymerase Chain Reaction-Based Method for Generating a Site Saturation Mutant Library. Anal Biochem (2014) 449:90–8. doi: 10.1016/j.ab.2013.12.002 24333246

[44] Ou J Zhou Z Dai R Zhang J Zhao S Wu X. V367F Mutation in SARS-CoV-2 Spike RBD Emerging During the Early Transmission Phase Enhances Viral Infectivity Through Increased Human ACE2 Receptor Binding Affinity. J Virol (2021) 95:e0061721. doi: 10.1128/JVI.00617-21 34105996 PMC8373230

[45] Hsieh C-L Goldsmith JA Schaub JM DiVenere AM Kuo H-C Javanmardi K. Structure-Based Design of Prefusion-Stabilized SARS-CoV-2 Spikes. Science (2020) 369:1501–5. doi: 10.1126/SCIENCE.ABD0826 PMC7402631 32703906

[46] Rathore U Purwar M Vignesh VS Das R Kumar AA Bhattacharyya S. Bacterially Expressed HIV-1 Gp120 Outer-Domain Fragment Immunogens With Improved Stability and Affinity for CD4-Binding Site Neutralizing Antibodies. J Biol Chem (2018) 293:15002–20. doi: 10.1074/jbc.RA118.005006 PMC6166733 30093409

[47] Hamborg L Granata D Olsen JG Roche JV Pedersen LE Nielsen AT. Synergistic Stabilization of a Double Mutant in CI2 From an in-Cell Library Screen. bioRxiv (2020) 2020.12.01.406082. doi: 10.1101/2020.12.01.406082

[48] Johansson KE Lindorff-Larsen K Winther JR. Global Analysis of Multi-Mutants to Discover Stabilizing Amino Acid Substitutions. bioRxiv (2020) 2020.12.03.408732. doi: 10.1101/2020.12.03.408732

[49] Zutz A Nielsen LH Pedersen LE Kassem MM Papaleo E Koza A. Nielsen AT. A Dual-Reporter System for Investigating and Optimizing Protein Translation and Folding in E. Coli. bioRxiv (2020) 2020.09.18.303453. doi: 10.1101/2020.09.18.303453 PMC8526717 34667164

[50] Li D von Schaewen M Wang X Tao W Zhang Y Li L. Altered Glycosylation Patterns Increase Immunogenicity of a Subunit Hepatitis C Virus Vaccine, Inducing Neutralizing Antibodies Which Confer Protection in Mice. J Virol (2016) 90:10486–98. doi: 10.1128/JVI.01462-16 PMC5110194 27630242

[51] Hutter J Rodig JV Hoper D Seeberger PH Reichl U Rapp E. Toward Animal Cell Culture-Based Influenza Vaccine Design: Viral Hemagglutinin N-Glycosylation Markedly Impacts Immunogenicity. J Immunol (2013) 190:220–30. doi: 10.4049/JIMMUNOL.1201060 23225881

[52] Boune S Hu P Epstein AL Khawli LA. Principles of N-Linked Glycosylation Variations of IgG-Based Therapeutics: Pharmacokinetic and Functional Considerations. Antibodies (Basel Switzerland) (2020) 9:22. doi: 10.3390/ANTIB9020022 PMC7345016 32532067

[53] Pollet J Chen W-H Versteeg L Keegan B Zhan B Wei J. SARS-CoV-2 RBD219-N1C1: A Yeast-Expressed SARS-CoV-2 Recombinant Receptor-Binding Domain Candidate Vaccine Stimulates Virus Neutralizing Antibodies and T-Cell Immunity in Mice. bioRxiv (2020). doi: 10.1101/2020.11.04.367359 PMC8054496 33847226

[54] Consortium AA. Structural and Functional Comparison of SARS-CoV-2-Spike Receptor Binding Domain Produced in Pichia Pastoris and Mammalian Cells. Sci Rep 2020 101 (2020) 10:1–18. doi: 10.1038/s41598-020-78711-6 PMC7732851 33311634

[55] Pino M Abid T Pereira Ribeiro S Edara VV Floyd K Smith JC. A Yeast Expressed RBD-Based SARS-CoV-2 Vaccine Formulated With 3M-052-Alum Adjuvant Promotes Protective Efficacy in Non-Human Primates. Sci Immunol (2021) 6:eabh3634. doi: 10.1126/sciimmunol.abh3634 34266981 PMC9119307

[56] Dalvie NC Tostanoski LH Rodriguez-Aponte SA Kaur K Bajoria S Kumru OS. A Modular Protein Subunit Vaccine Candidate Produced in Yeast Confers Protection Against SARS-CoV-2 in Non-Human Primates. bioRxiv (2021) 2021.07.13.452251. doi: 10.1101/2021.07.13.452251

[57] Walls AC Fiala B Schäfer A Wrenn S Pham MN Murphy M. Elicitation of Potent Neutralizing Antibody Responses by Designed Protein Nanoparticle Vaccines for SARS-CoV-2. Cell (2020) 183:1367–82.e17. doi: 10.1016/j.cell.2020.10.043 33160446 PMC7604136

[58] Cohen AA Gnanapragasam PNP Lee YE Hoffman PR Ou S Kakutani LM. Mosaic Nanoparticles Elicit Cross-Reactive Immune Responses to Zoonotic Coronaviruses in Mice. Science (2021) 371:735–41. doi: 10.1126/science.abf6840 PMC7928838 33436524

[59] Brouwer PJM Brinkkemper M Maisonnasse P Dereuddre-Bosquet N Grobben M Claireaux M. Two-Component Spike Nanoparticle Vaccine Protects Macaques From SARS-CoV-2 Infection. Cell (2021) 184:1188–1200.e19. doi: 10.1016/J.CELL.2021.01.035 33577765 PMC7834972

[60] Kang Y-F Sun C Zhuang Z Yuan R-Y Zheng Q Li J-P. Rapid Development of SARS-CoV-2 Spike Protein Receptor-Binding Domain Self-Assembled Nanoparticle Vaccine Candidates. ACS Nano (2021) 15:2738–52. doi: 10.1021/ACSNANO.0C08379 33464829

[61] Ma X Zou F Yu F Li R Yuan Y Zhang Y. Nanoparticle Vaccines Based on the Receptor Binding Domain (RBD) and Heptad Repeat (HR) of SARS-CoV-2 Elicit Robust Protective Immune Responses. Immunity (2020) 53:1315–30.e9. doi: 10.1016/J.IMMUNI.2020.11.015 33275896 PMC7687490

[62] Marcandalli J Fiala B Ols S Loré K Perez L Correspondence NPK. Induction of Potent Neutralizing Antibody Responses by a Designed Protein Nanoparticle Vaccine for Respiratory Syncytial Virus In Brief A Computationally Designed Self-Assembling Nanoparticle That Displays 20 Copies of a Trimeric Viral Protein Induces Potent Neutralizing Antibody Responses. Cell (2019) 176:1420–31.e17. doi: 10.1016/j.cell.2019.01.046 30849373 PMC6424820

[63] Kanekiyo M Wei C-J Yassine HM McTamney PM Boyington JC Whittle JRR. Self-Assembling Influenza Nanoparticle Vaccines Elicit Broadly Neutralizing H1N1 Antibodies. Nature (2013) 499:102–6. doi: 10.1038/nature12202 PMC8312026 23698367

