# Supplementary material for: A Stabilized, Monomeric, Receptor Binding Domain Elicits High-Titer Neutralizing Antibodies Against All SARS-CoV-2 Variants of Concern
Source: Front Immunol. 2021 Dec 9;12:765211. doi: 10.3389/fimmu.2021.765211 (PMC8695907; doi:10.3389/fimmu.2021.765211)
Supplement: Supplementary file 1 [file DataSheet_1.docx]

**Supplementary Materials**


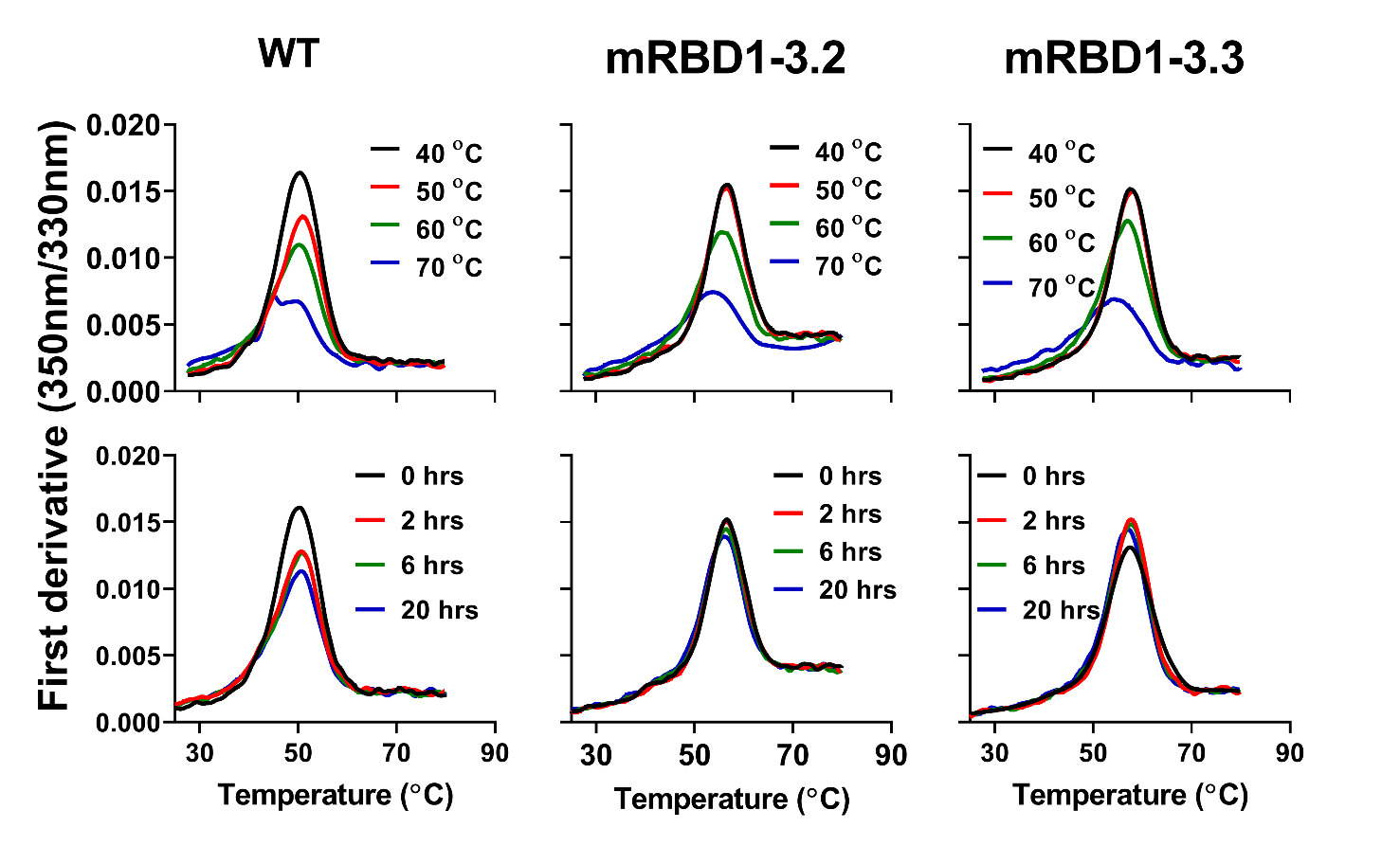


**Supplementary Figure S1: Thermal tolerance of WT and multimutants assayed using DSF.** The proteins were heated for two hours at different temperatures (top panel) or at 50 ^ᴼ^C for different duration (bottom panel). The peak height provides information on the amount of folded protein left after thermal stress. The mutants showed better thermal tolerance than WT when incubated at 40 ^ᴼ^C, 50 ^ᴼ^C and 60 ^ᴼ^C for two hours. The mutants were also resistant to unfolding when incubated at 50 ^ᴼ^C for different time points and showed no significant reduction in the peak height compared to unstressed controls.


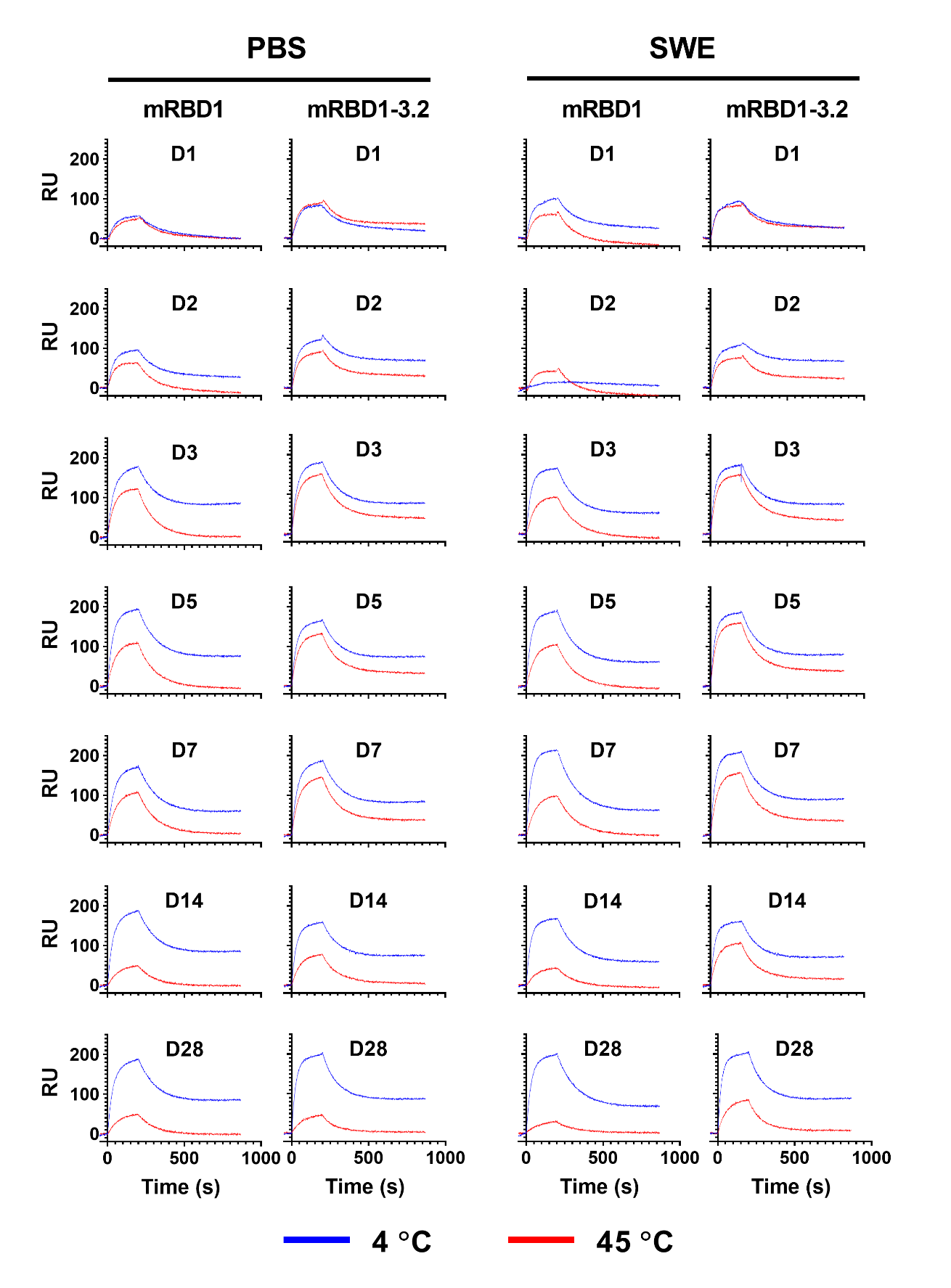


**Supplementary Figure S2: Characterization of mRBD1 and mRBD1-3.2** **following extended incubation at 45 °C.** mRBD and mRBD1-3.2 (0.2 mg/ml) in solution (PBS), without or with SWE adjuvant subjected to 45 °C incubation as a function of time (1-28 Days). At the indicated timepoint, samples were stored at 4°C until analysis.The binding to ACE2-hFc was performed with 100 nM RBD analyte. 800RU ACE2-hFc was immobilized.


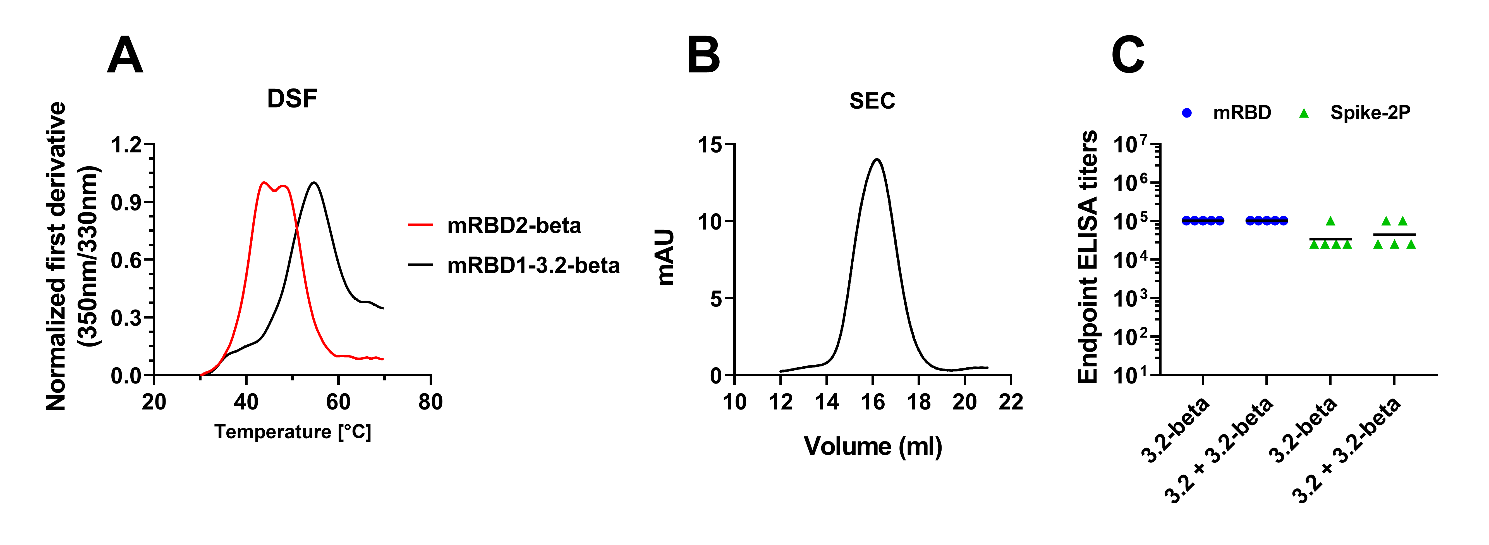


**Supplementary Figure S3: Additional characterization of mRBD1-3.2-beta.** (A) Comparison of thermal stability of mRBD2-beta and mRBD1-3.2-beta. (B) SEC profile of purified mRBD1-3.2-beta. (C) mRBD1-3.2-beta as well as a cocktail formulation of mRBD1-3.2 and mRBD1-3.2-beta generated significant antibody titers against mRBD and Spike-2P.


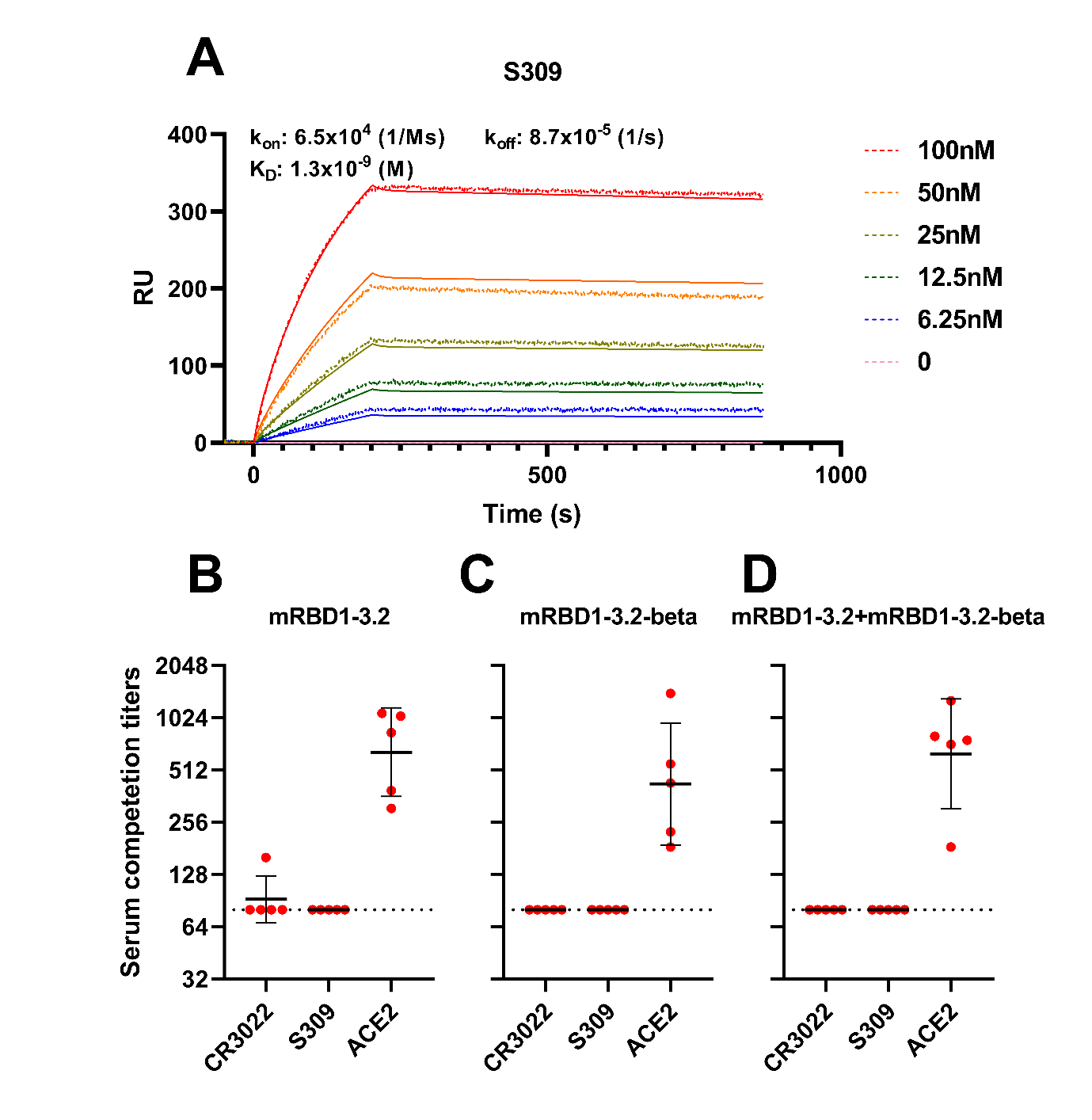
 **Supplementary Figure S4: Binding of S309 MAb with mRBD1-3.2 and competition of mice sera with monocolonal antibodies and ACE-2 hFc.** (A) Binding of mRBD1-3.2 with S309 antibody monitored by SPR. Competition binding titers for sera from mice immunized with (B) mRBD3.2, (C) mRBD3.2-beta and (D) formulation of mRBD3.2 and mRBD3.2-beta. Competition titers <80 were assigned a value of 80 for display purposes, this is indicated by a dotted line. Significant competition was only observed against ACE2. Kruskal-Wallis with Dunn's multiple comparisons tests were performed for paired comparisons of competition titers. * and ** indicates P< 0.05 and < 0.01 respectively.


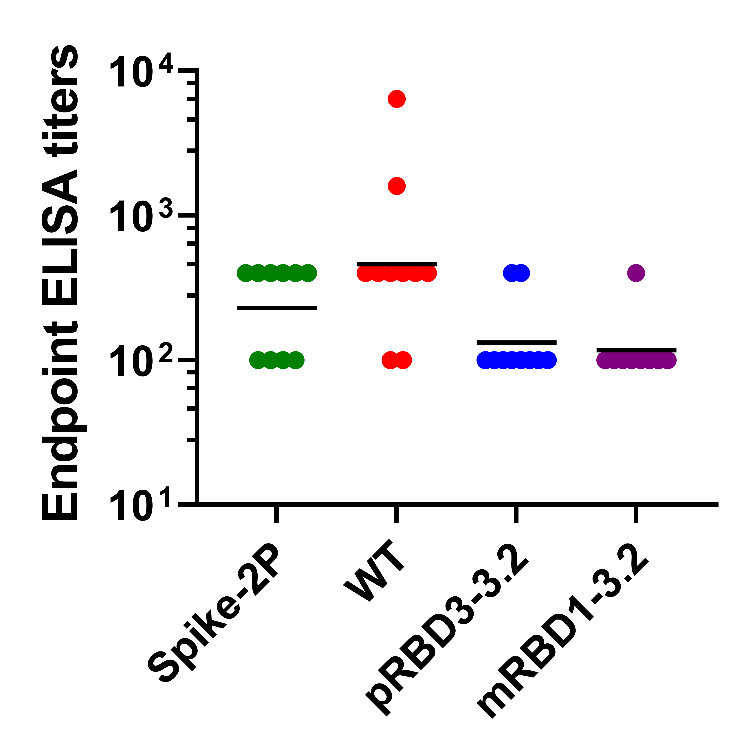


**Supplementary Figure S5: Binding of Spike-2P, WT RBD, pRBD3-3.2 and mRBD1-3.2 with HCS.** Antibodies present in HCS bind to spike, WT RBD and stabilized variants to a similar extent. No statistically significant difference in binding of HCS to Spike-2P and RBDs was observed. Kruskal-Wallis with Dunn's multiple comparisons tests with Spike-2P were performed for paired comparisons of binding titers.
